# Control of Pumps of Water Supply Network under Hydraulic and Energy Optimisation Using Artificial Intelligence

**DOI:** 10.3390/e22091014

**Published:** 2020-09-11

**Authors:** Jan Studziński, Andrzej Ziółkowski

**Affiliations:** Systems Research Institute of Polish Academy of Sciences, Pl 01-447 Warsaw, Poland

**Keywords:** water supply network, pump control algorithms, hydraulic optimisation, cost optimisation, genetic algorithm, fuzzy sets

## Abstract

This article presents several algorithms for controlling water supply system pumps. The aim of having control is the hydraulic optimisation of the network, i.e., ensuring the desired pressure in its recipient nodes, and minimising energy costs of network operation. These two tasks belong to the key issues related to the management and operation of water supply networks, apart from the reduction in water losses caused by network failures and ensuring proper water quality. The presented algorithms have been implemented in an Information and Communications Technology (ICT) system developed at the Systems Research Institute of the Polish Academy of Sciences (IBS PAN) and implemented in the waterworks GPW S.A. in Katowice/Poland.

## 1. Introduction

The Systems Research Institute has been developing Information Technology (IT) systems to support the management of municipal water supply companies for several years. As a result of these works, an ICT system has been developed for modelling and optimising water supply networks [1]. It is a tool which, at the design stage, provides an opportunity to verify the adopted design assumptions and check the behaviour of the water network after its possible expansion, while at the operation stage, it provides an opportunity to determine the actual flows and pressures in the network and scenarios for its optimisation and control. The system enables rational management of the water supply network in the field of flow and pressure regulation, selection of pipeline diameters, pump operation, and detection of anomalies and abnormal conditions in networking. Correct operation of the water supply network and strategic planning of investments on it requires conducting analyses on the basis of the information available about its operation. This is possible with the use of a hydraulic model of the water network, which allows for:

- Analysis of exceptional and special situations, e.g., water intake for fire protection purposes, assessment of the effects of changing the direction of water transport in case of failure and shutdown of selected pipelines;
- Analysis of network efficiency in terms of planned expansion, changes related to water distribution, increased water intake (e.g., seasonal recipients), network modernisation (e.g., revitalisation of selected pipes with high unit resistance);
- Optimisation of network operation in order to minimise operating costs, pressure management in individual pressure zones and operation of zone intakes and pumping stations;
- Outlining investment activities related to the improvement of water quality and reliability of water supply.

The need to reduce the costs of functioning of water networks (by reducing energy consumption, reducing water losses, eliminating water theft, and minimising the costs of water treatment, among others), increasing the efficiency of management of the water supply system in use, and supporting the investment process are the problems that must be faced by an enterprise that has a permit to operate in the field of communal water supply.

Water supply companies most often collect information about their water network in the database of a Geographic Information System (GIS) installed in the waterworks. This database contains detailed information about the structure of the network, connections of its pipelines, and parameters describing particular elements and objects mounted on the network. These data enable the formulation of a hydraulic model describing the water network. On the other hand, calibration of the hydraulic model is possible by means of measurement data obtained from the Supervisory Control and Data Acquisition (SCADA) monitoring system assembled in the waterworks [2,3].

The ICT system, in which the control algorithms presented below were implemented, was developed in IBS PAN. The IT system has a modular structure and consists of a GIS and SCADA, AMR (Automated Meter Reading) and billing monitoring systems, and of a MOSKAN-W calculation system, containing a hydraulic model of the water supply network and control algorithms using this model and supporting the water network management. The systems GIS, SCADA, AMR, and a billing system are the solutions of different IT companies adapted to the needs of the ICT system, while the MOSKAN-W system is an original solution of IBS PAN.

Below we will show four examples of using the MOSKAN-W system to control the speed of a pumping station pumping water into the water network at the water supply company GPW S.A. in Katowice/Poland. In example 1, the load on the water supply network is reduced, which makes it possible to reduce the rotation speed of the pump, while ensuring the desired pressure at the exit pipelines of the pumping station. In example 2, when the load on the water supply network is fixed, the pressure at the exit from the pumping station should be increased, which requires increasing the pump speed. Example 3 concerns the regulation of the outlet pressure at the pumping station by changing the pump speed and changing the diameters of the water pipes concerned. Finally, example 4 provides the desired outlet pressure at the pumping station by adjusting the pump speed and at the same time, by minimising the energy costs of the pumping station and the costs of treating the water pumped into the water supply network.

Examples 1 and 4 deal with the issue of energy-efficient operation of the water supply network, which is one of the key management problems of a water supply company. The control of changing the speed of pumps in pumping stations installed on the water supply network, sewage network, and sewage treatment plant is the main factor for increasing electricity savings in a water supply company [4], because these devices are the main consumers of this energy and they are primarily responsible for its usage [5].

For solving the tasks of managing the water supply network, artificial intelligence methods are used in the MOSKAN-W system, with fuzzy sets and a genetic algorithm of multicriteria optimisation applied in the examples discussed.

Artificial intelligence is a branch of computer science that uses mathematical modelling and optimisation methods based on the simulation of natural human behaviour, including above all the functioning of the brain, evolutionary processes taking place in the body, and verbal communication. These methods use mainly neural networks [6], fuzzy logic [7], and evolutionary calculations [8].

In the examples discussed below, we do not use neural networks; the fuzzy sets technique is used to define the variation ranges of variables controlled in the optimisation algorithm, while the modified genetic optimisation algorithm is used to solve the formulated optimisation tasks.

## 2. General Characteristics of the MOSKAN-W System

The MOSKAN-W system is a key module of the IT system, which also consists of a GIS and SCADA and Automated Meter Reading (AMR) monitoring and billing systems. Calculation data for the MOSKAN-W system can be loaded from the GIS database and prepared in the form of imported text files or created in an interactive mode. The application calculation kernel is Epanet2 software [9,10] developed by Water Supply and Water Resources Division of the U.S. Environmental Protection Agency—EPA [11]. Hydraulic losses in the network are calculated on the basis of Hazen–Williams, Darcy–Weisbach, or Chezy–Manning calculation formulas. The hydraulic calculations of the network model use the global gradient method [12], which is a modified version of the Newton–Raphson method [13].

The data concerning the water network and gathered in the GIS database are constantly updated. Most often they are too detailed from the point of view of the network modelling and optimisation. Too much detail in the network model does not determine its greater correctness, but only unnecessarily complicates hydraulic calculations and extends the calculation time.

In addition to the data describing the network and collected in the GIS database, measurement data are also needed to enable model calibration. The measurement data can be gathered during measurement campaigns carried out with rented equipment or with measurement devices permanently installed at many points in the network. The second solution, although more expensive, has many advantages, as the constantly gathered and recorded measurement data allow for quick updating of the model parameters after changes made to the network and the carrying out of the periodic recalibration of the network hydraulic model.

The integration of MOSKAN-W with the GIS simplifies the process of preparing the data for modelling. The integration of both systems makes it possible to select a specific part of the network in the GIS and generate data for the MOSKAN-W system to model only this part. The measurement data of the water consumption by network users are extracted from the billing system and the AMR system, pre-processed in the GIS, and then, made available to the MOSKAN-W system as aggregated tables. In turn, the measurement data from the measurement points located in the network are imported by the MOSKAN-W system directly from the SCADA system database.

## 3. Control of Pumps of the Water Supply Network

The control of pumps installed in the pumping stations of the water supply network is intended to provide network users with the required amount of water and to ensure that the network’s recipient nodes are sufficiently pressurised. Pumps control is currently regulated in water supply companies essentially in two ways.

The first way is to establish the rules for controlling the pumps depending on the expected network load [14,15]. In general, three repeated load phases can be distinguished in the operation of municipal water supply systems: high load in the morning and afternoon hours, medium load in the noon hours, and low load in the night hours [16]. Each communal water network is characterised by load characteristics typical for itself, which can be determined by averaging loads over several days, divided into working days and holidays (weekends), and based on these characteristics, schedules of pumping station operation can be developed [17,18,19,20].

The second way is to make the operation of the pumps dependent on current pressure measurements of the network’s recipient nodes by using automatic control systems in the pumping stations. The pressure drop, which is signalled by the water meters installed at the recipient connections to the water network, controls the pressure level and flow value generated by the pumps [17]. A pressure drop at the network end nodes usually means an increase in load, although it is often also a sign of emergency states and water leaks caused by them.

At the same time, pump control algorithms are developed with the use of optimisation algorithms, which define the criteria for minimising energy consumption and the operating costs of the water network [21,22,23], the number of switching on and off of individual pumps in multi-pump stations to extend their service life [24,25,26], and the speed of changes in pump operation to avoid hydraulic impacts in the network, which may cause failures [27]. It should be noted that similar pump control tasks using optimisation algorithms are also solved in other key facilities of water supply companies, i.e., sewage networks and sewage treatment plants [28,29].

In optimisation calculations, multicriteria optimisation algorithms are usually used, including in particular, genetic algorithms [30,31,32,33], while for network modelling, the Epanet2 program [14,34] is applied, using in hydraulic calculations the global gradient method.

The control algorithms determined in this way, usually tested and showing high efficiency in laboratory conditions, are not implemented in water supply companies due to their high level of complexity and also high costs, generated primarily by the prices of measuring equipment of the monitoring systems installed on water networks, while appropriately dense monitoring systems are necessary for the correct calibration of the network’s hydraulic models.

## 4. Fuzzy Sets and Genetic Algorithm

Optimisation tasks occurring in the management of water supply networks are, in most cases, multicriteria optimisation tasks. Fuzzy sets and fuzzy logic are a convenient mathematical apparatus for describing such tasks. All criteria and constraints can then be treated as fuzzy values, forming the fuzzy set. A certain range of values of a defined quality criterion may meet fully accepted requirements (preferred values), but a wider range of values of the criterion, where these requirements are met to a lesser extent (permissible values), may also be given. There is also a range of values of a criterion where the requirements are not met at all. The use of fuzzy sets facilitates unification of the problem description, realisation of the interface of the created PC program, and development of optimisation algorithms.

**Figure 1:**
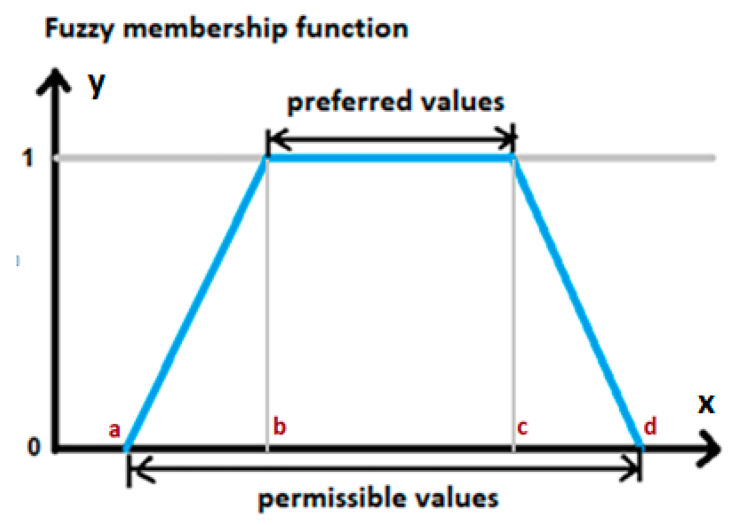
Trapezoidal affiliation function defined in fuzzy sets.

The criteria and limitations represented by fuzzy sets can be approximated by a simple trapezoidal function defined by giving four numbers (Figure 1) [35].

The trapezoidal function in Figure 1 defines the degree of belonging to the values set *Criterion fulfilled*, on which the function defining the formulated quality criterion is determined. For preferred values, the degree of belonging is 1, and for values outside the range of permissible values, it is 0. For other values of the criterion (permissible values), the affiliation function takes the values from the range (0,1).

Determining the optimal solution consists of finding such values of control variables, for which the degree of belonging of the value of the quality criterion to the values set *Criterion fulfilled* will be 1 for all criteria defined in the optimisation task, and if this is not available, then determining such values of control variables for which the degree of belonging to the worst criterion will be the highest.

When performing the optimisation task, we look for solutions in a set of *n* control variables:

(1) $$<x_{1},x_{2},\ldots ,x_{n}>\;\in R^{n\;}:\left\{x_{1}^{min}\leq x_{1}\leq x_{1}^{max},x_{2}^{min}\leq x_{2}\leq x_{2}^{max},\ldots ,x_{n}^{min}\leq x_{n}\leq x_{n}^{max}\right\}$$

For each of the *m* criteria defined, the value of the criterion function can be calculated from the equation:

(2) $$y_{j}=\;f_{j}(x_{1},x_{2},\ldots ,x_{n})\;for\;j=1,2,\ldots ,m$$

The criterion functions *f_j_* are defined independently for each optimisation task and they are not in analytical form, but after calculating the function for the point $(x_{1}$,$\;x_{2}$,…,$x_{n}),$ it is possible to determine its value.

We do not have an analytical form of the function *f_j_*, but after calculating the function for the point $(x_{1}$,$\;x_{2}$,…,$x_{n}),$ it is possible to determine its value.

Taking the trapezoidal affiliation function (see Figure 1) for the fuzzy variable *Criterion j fulfilled*, we can determine the degree of belonging for the variable *j*:

(3) $$P_{j}=\;\left\{\begin{array}{c} \begin{array}{c} \begin{array}{c} \begin{array}{c} \;0\;if\;y_{j\;}\leq \;a_{j}\\ \frac{y_{j}-\;a_{j}}{b_{j}-\;a_{j}}\;if\;a_{j\;}<\;y_{j\;}<\;b_{j} \end{array}\\ \;1\;if\;b_{j\;}\leq \;y_{j\;}\leq \;c_{j} \end{array}\\ \frac{d_{j}-\;y_{j}}{d_{j}-\;c_{j}}\;if\;c_{j\;}<\;y_{j\;}<\;d_{j} \end{array}\\ \;0\;if\;y_{j\;}\geq \;d_{j} \end{array}\right\}$$

and the degree of belonging to the fuzzy variable *All criteria fulfilled*:

(4) $$P=\min(P_{1},P_{2},\ldots ,P_{m})$$

the value of which will be maximised.

Optimisation software used in practice should facilitate interpretation and verification of results. This can be achieved through an appropriate presentation of the results in the optimisation process. During the optimisation process, the degree of fulfilment of all criteria and the values of the control (decision) variables of the best solution found are shown.

Gradient optimisation methods fail in difficult optimisation tasks, which include water supply network management tasks. Such tasks can be solved with the use of genetic optimisation methods [28,36]. Algorithms of genetic optimisation guarantee the approach to optimal solutions with a relatively large number of calculations of functions defining quality criteria, but do not guarantee finding an optimal solution.

The principle of operation of genetic algorithms can best be explained by analogy to the phenomena of population adaptation to the environment in biological systems. The population is made up of individuals whose characteristics are determined by a set of genes (genotype). Depending on their characteristics, individuals are better or worse adapted to the environment. During the evolutionary adaptation of the population to the environment, the following phenomena occur:

- Some of the worst adapted individuals are removed from the population (the extinction of the weakest);
- The population is supplemented by new individuals whose genotypes are created by mixing parental genotypes or changing gene parts (mutations).

A genotype is a set of all control variables describing the water supply network under investigation, a gene is one parameter in one pipeline of the network, and an individual is each set of values of all control variables. The calculated value of the quality criterion determines the degree of adaptation. The process of evolutionary adaptation of the population to the environment allows the finding of solutions that best meet the adopted quality criteria (best adapted individuals).

Optimisation calculations are carried out in two stages. In the first stage, different genotypes are randomly generated, which are added to the population until a given population size is reached. At this stage, genotypes are not removed (no extinction of the weakest individuals).

In the second stage of calculation, new genotypes are added in each iteration and the least adapted genotypes are removed. Thus, the size of the population is constant and its overall adaptation cannot decrease.

In addition, each gene can be mutated with some probability. This means that in a new genotype, part of the genes may not come from population genotypes, but may be generated randomly.

In order to speed up the process of finding optimal solutions, a modification of the classical genetic algorithm has been developed, which uses information about the so-called critical criteria, limiting the search area based on data collected from the group of individuals which best meet the critical criteria. A set of individuals described by a set of genes is generated, and each gene corresponds to the value of one independent variable. The worst-adapted individuals are removed from the population, which in the least degree belong to the fuzzy set *All criteria fulfilled*.

The developed optimisation algorithm consists of the following steps:

1. Random generation of genotypes of all individuals in the population.
2. Performing simulations on the basis of genotype data, i.e., calculation and storage of values of criteria variables and degrees of belonging to fuzzy variables, successively for all individuals in the population.
3. Interruption of calculation if the degree of belonging to the fuzzy variable *All criteria fulfilled* is 1 or if the user has requested their interruption.
4. Selection of the best adapted individuals from the whole population.
  The basis for selection may be the overall result of the individual or only the result of the critical criterion.
5. Determination of ranges of variability of independent (control) variables in the selected group of individuals.
6. Determination of a narrower search area on the basis of previously determined ranges of variability.
  The search area may be additionally narrowed down by considering only independent variables strongly correlated with the results of the critical criterion and by omitting variables that do not have a significant impact on the values of the critical criterion. This operation requires performing additional calculations on the data stored for the whole population.
7. Random generation of a new group of individuals with parameters from a narrower search area and addition of the group to the population.
8. Move to step 2.

## 5. Tasks for Hydraulic Optimisation of the Water Supply Network

The ICT system developed at the Systems Research Institute contains a water network hydraulic model and pump control algorithms designed for the waterworks company GPW S.A. in Katowice/Poland [2,3]. The water network of this enterprise covers the entire area of the Upper Silesian Voivodship, with an area of about 12,300 km^2^. It is a distribution network supplying drinking water to the cities of Upper Silesia and their individual water supply systems.

In this section, four optimisation tasks carried out by this system will be presented. The tasks concern the hydraulic optimisation of a selected fragment of the investigated water supply network—the water treatment plant and pumping station Bibiela.

### 5.1. Optimal Pump Speed for Changed Water Consumption

This is an example of using the ICT system to solve problems related to the control of pumping stations on the water supply network. The task is to control the pump speed in the pumping station in such a way that after changing the value of water consumption in selected network nodes, the required pressure head values are provided in these nodes.

This is a representation of a situation where, at night, the water intake by the water supply network decreases in comparison with the daily intake and then, it is advisable to reduce the pressure in the pumping station in order to avoid unnecessary generation of costs.

**Figure 2:**
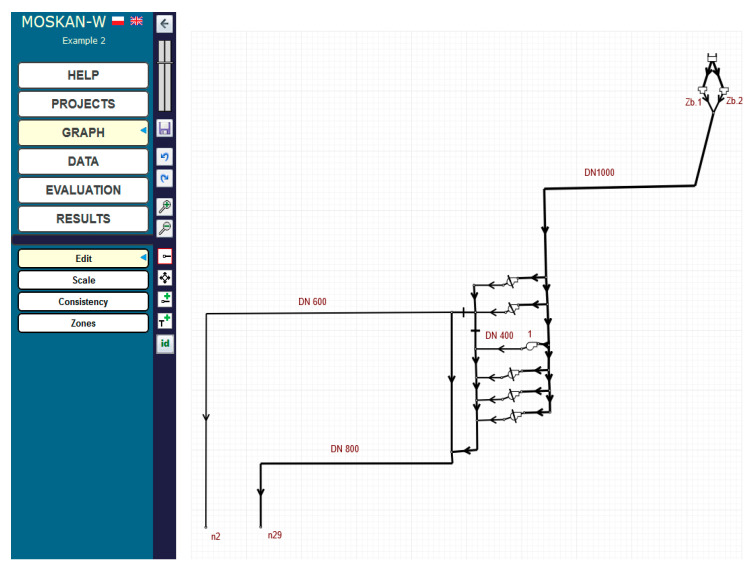
Diagram of the Bibiela pumping station.

Figure 2 shows a diagram of the Bibiela pumping station, which is one of eight source pumping stations at the GPW S.A. company. In the figure, the situation is presented when pump 1 pumps water into the mains of the water network from two tanks. In fact, there are two pump units in the Bibiela pumping station. The first unit of pumps is in the water intake station; the drawn water is then treated and directed to the tanks, from which the second pump’s unit pumps it into the mains. In our example, we control pump 1 in the second unit of pumps, so the figure does not take into account the other objects of the pumping station. It is worth mentioning that the configuration of the pump in Figure 2 and the other upcoming figures is symbolic, taken by the software, and is not the actual situation, as the directions of the inlet and outlet flows of the pump are really the inverse.

Pump 1, working at a speed equal to 0.76 of the nominal speed, ensures the pressure head of 11.8 m in node n2 at the water consumption of 334 m^3^/h and the same pressure head in node n29 at the water consumption of 866 m^3^/h. When determining the acceptable values range for the pump speed as a control variable, we take the value 1 in the optimisation algorithm as the nominal speed value for clearer interpretation of data and calculation results, while in the calculation, the pump speed takes the real value, different for different calculation cases.

The task now is to find a pump speed that will ensure an adequate pressure head in the relevant nodes with significantly reduced water consumption values to 226 m^3^/h for node n2 and to 650 m^3^/h for node n29.

**Figure 3:**
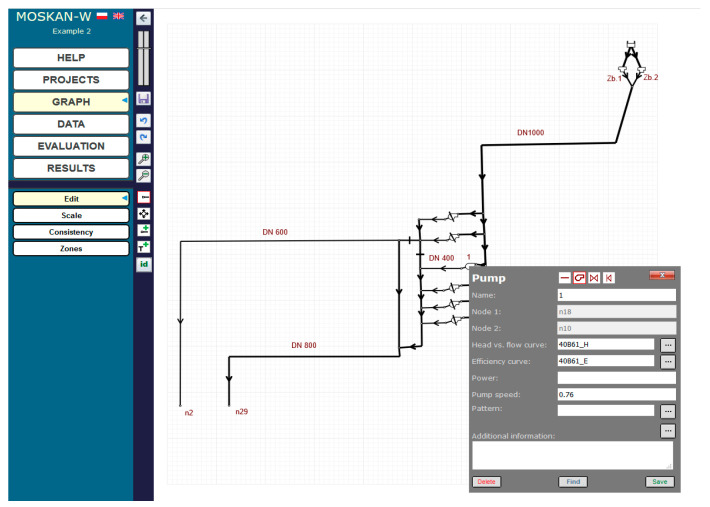
Parameters of the controlled pump 1.

**Figure 4:**
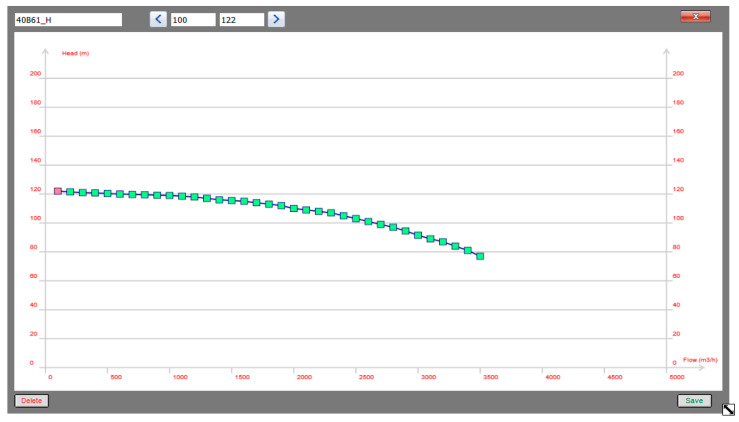
Pressure head characteristics of pump 1: flow vs. pressure head.

**Figure 5:**
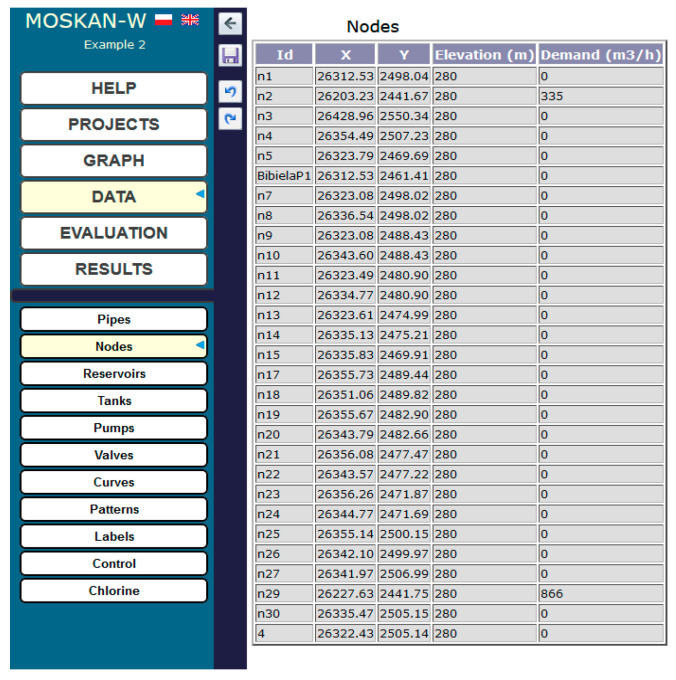
Reduced values of water consumption in the Bibiela pumping station.

Figure 3 shows the parameters of pump 1 used in the pumping station. The pressure head and flow dependence are determined by the pump characteristics shown in Figure 4, while Figure 5 shows the new water consumptions in nodes n2 and n29.

**Figure 6:**
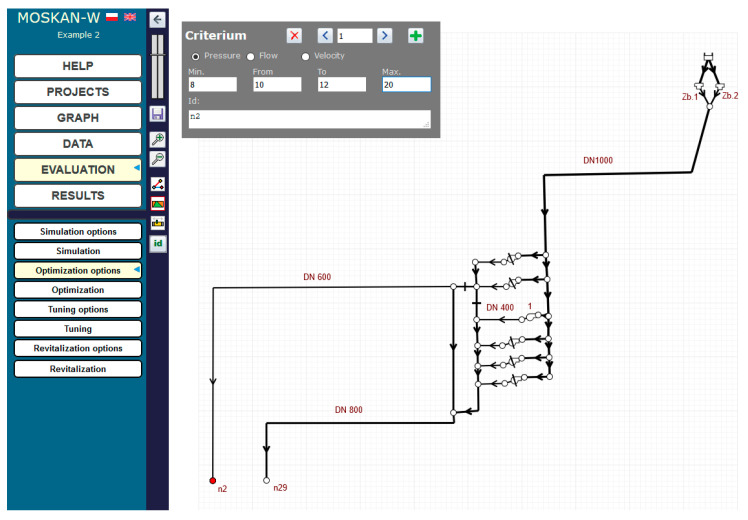
Definition of pressure head variation ranges for nodes n2 and n29.

**Figure 7:**
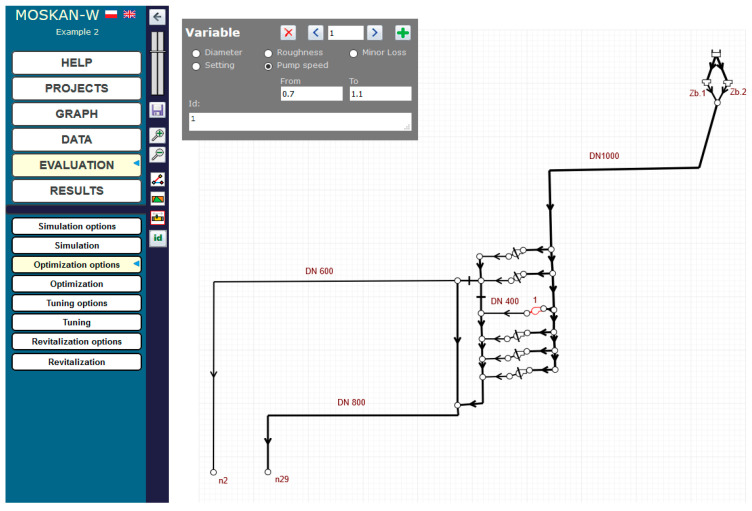
Definition of the control variable (pump 1 speed) variation range (Task 5.1).

The solution of the problem using the algorithm presented is to define the optimisation option, i.e., to define the quality criteria and control variables. The quality criteria defined in the task are the pressure head values in nodes n2 and n29 at the exit from the pumping station, for which preferred (10–12 m) and permissible (8–20 m pressure head values should be defined (Figure 6). The control variable is the speed of pump 1, for which the speed range (0.7–1.1) should be assumed and for which the solution of the task will be sought (Figure 7).

In this example, we have two defined criteria: reaching the required pressure head value at node n2 and at node n29 of the network. The required values were determined as a fuzzy variable described by a trapezoidal affiliation function defined by giving four numerical values that determine the trapezoid.

**Figure 8:**
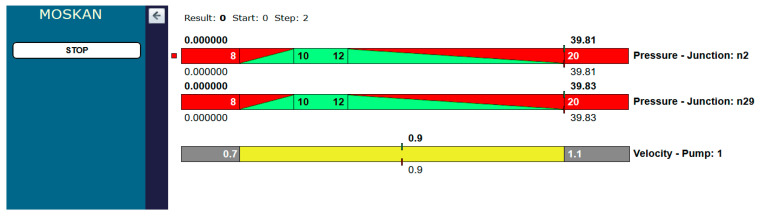
Start of the optimisation process (Task 5.1).

**Figure 9:**
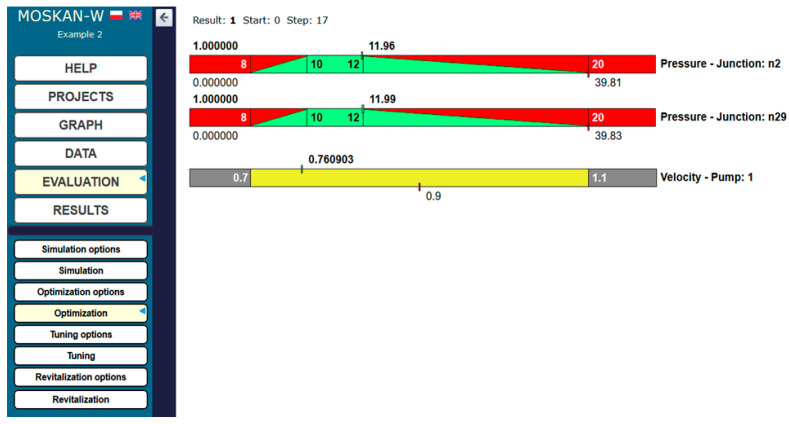
Final results of optimisation (Task 5.1).

Figure 8 shows the values of quality criteria and the value of the control variable at the beginning of the optimisation process, while Figure 9 shows the final results, when the algorithm found a satisfactory solution after 17 steps of calculation. The values of pressure heads in both the n2 and n29 nodes, in which the water consumption values decreased, are in the ranges of preferred values, and at the same time, the pump speed decreased to 0.76 of the nominal speed.

### 5.2. Optimal Pump Speed for Fixed Water Consumption

This is a task of hydraulic optimisation of the water supply network, taking into account the control of only the rotational speed of the pump in the source pumping station. This reflects the situation when there is unsatisfactory pressure in the network recipient nodes (user nodes) and the problem needs to be solved without investment outlays.

In this task, we again take care of the pumping station in Bibiela. The pressure head at nodes n2 and n29 is 1.13 m and it is too low according to the network operator, while the pump speed in the pumping station is 0.7 of the nominal value and is also relatively low.

The solution to the task is to increase the speed of the pump in such a way that the pressure head at selected network nodes, at the preset water consumption, reaches the preferred values, i.e., from the range of values (15–20 m).

**Figure 10:**
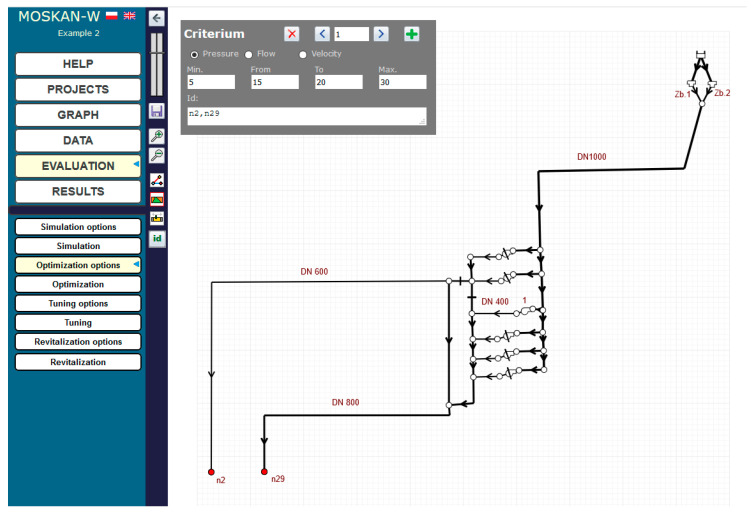
Definition of pressure head variation ranges for nodes n2 and n29 (Task 5.2).

**Figure 11:**
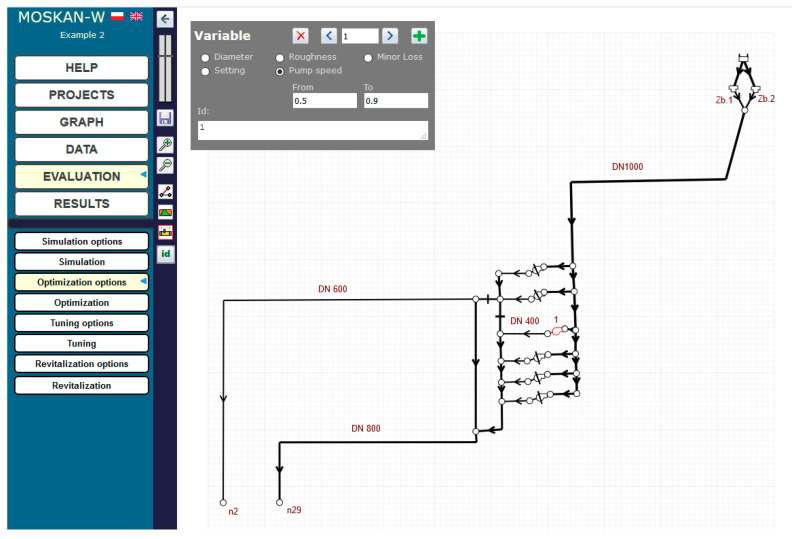
Definition of the control variable (pump speed) variation range (Task 5.2).

At the same time, the same preferred pressure values for both nodes are postulated, i.e., only one quality criterion is defined (Figure 10). The range (0.5–0.9) of the pump’s nominal speed is taken for the permissible speed values (Figure 11).

In this example, we have only one criterion defined jointly for two nodes n2 and n29 and this concerns the achievement of the required pressure head value. This is a convenience for the algorithm user, as the calculations are the same as if we had defined two independent criteria with the same parameters.

**Figure 12:**
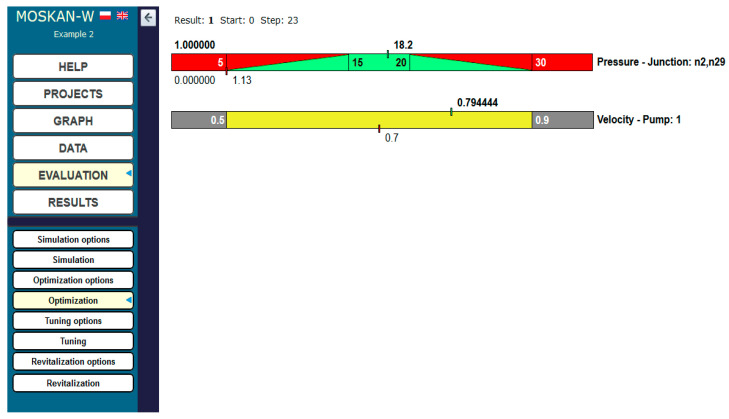
Optimisation results (Task 5.2).

In the optimum solution, the pressure head at both nodes of the network increased to 18.2 m and at the same time, the pump speed increased to 0.79 of its nominal value (Figure 12).

### 5.3. Optimal Pump Speed and Specified Pipe Diameters for Pressure Regulation in Specified Nodes

The next task concerns the hydraulic optimisation of the water supply network with the possibility of performing investment works related to the replacement of selected network pipelines with pipes of different, usually larger, diameter. The examined example is the Bibiela pumping station again.

The presented task often occurs in the case of old water supply networks, when the pipes become overgrown, increasing the hydraulic resistance of water flow in them, and in turn, the progressive rusting of old pipes increases their failure rate. Therefore, this task solves two key problems related to water supply network management at the same time: it improves the pressure in selected network nodes and reduces its failure rate, and consequently, reduces the water losses and network operating costs.

In this example, we again have two criteria, as in point 5.1, and they concern achieving the required pressure head values at node n2 and node n29, while the required pressure heads at both nodes are different.

**Figure 13:**
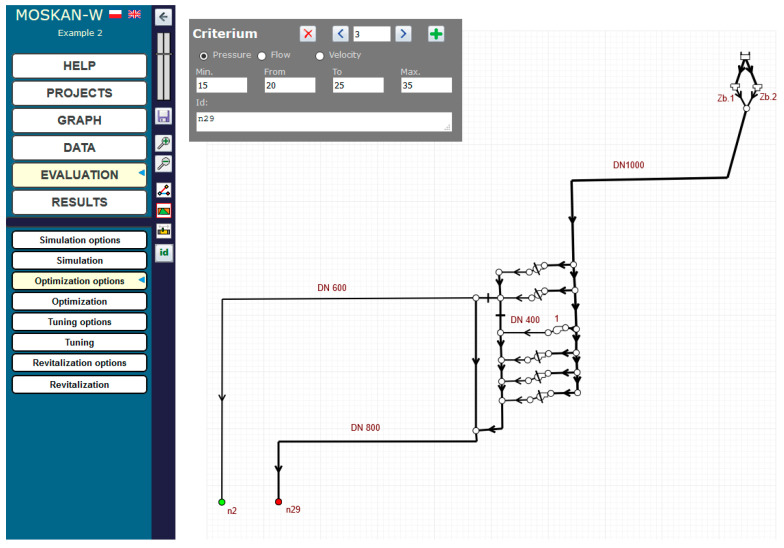
Definition of pressure head variation ranges for node n29 (Task 5.3).

**Figure 14:**
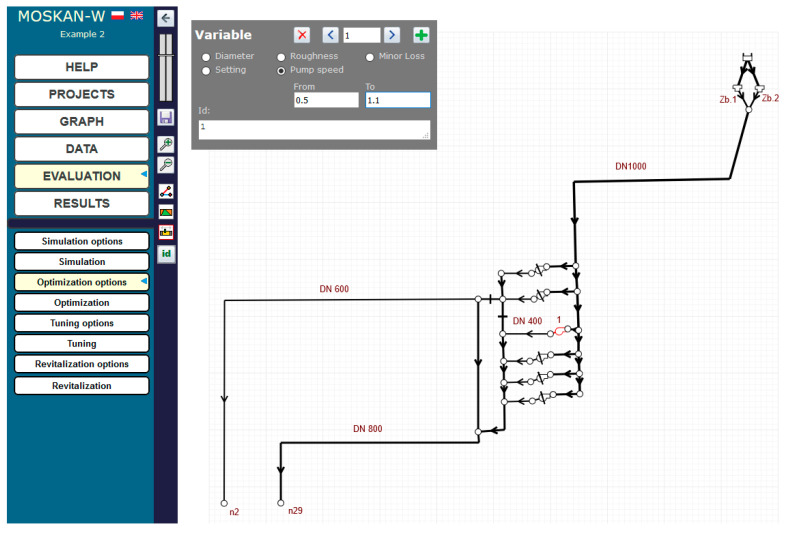
Definition of the control variable (pump l speed) variation range (Task 5.3).

**Figure 15:**
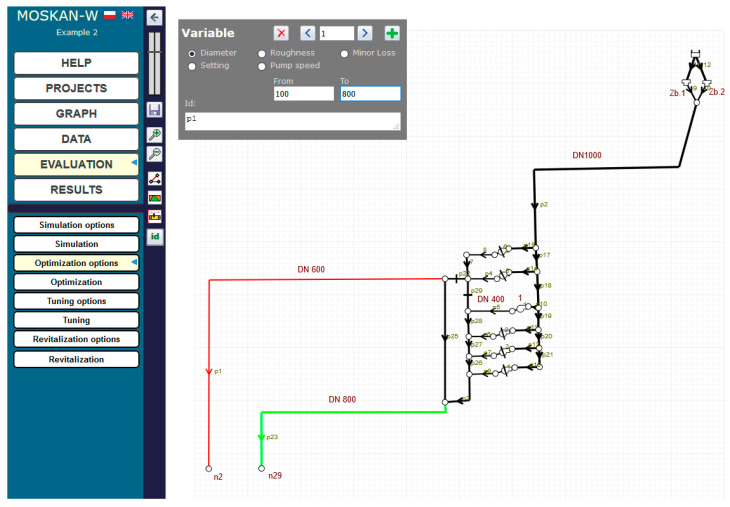
Definition of the control variable (pipe p1 diameter) variation range (Task 5.3).

Figure 13 shows the example of defining the quality criterion for node n29. For both nodes n2 and n29, separate criteria are defined, postulating different preferred pressure heads at both nodes, i.e., (15–20) m for n2 and (20–25) m for n29. Figure 14 and Figure 15 show the definition of variability ranges for the decision variables, i.e., for the speed of the pump 1 (values range (0.5–1.1)) and for the diameters of two network pipelines p1 and p23, while for both pipes the same acceptable range of diameter variability (100–800 mm) was determined.

**Figure 16:**
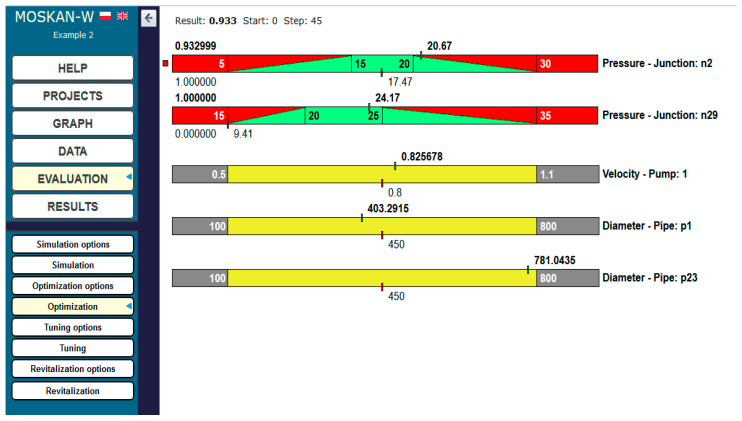
Optimisation results (Task 5.3).

The optimisation results are shown in Figure 16. At node n29, the pressure head increased from the initial value of 9.41 m to the preferred value of 24.17 m (increase by approx. 150%), while for pipe p23 leading to this node (green in Figure 15), it is proposed to change the diameter from 450 to 800 mm (an increase by approx. 80%; the optimisation algorithm determines the decision variables from continuous intervals; therefore, in the case of diameters, the obtained value should be rounded to the nearest value corresponding to the nominal diameter of the pipe).

At node n2, the pressure head value of 20.67 m was obtained, i.e., slightly exceeding the preferred values (by 3%) and being within the range of permissible values, while the diameter of pipe p1 (from 450 to 400 mm) can be reduced by approx. 13%.

The results concerning pressure head values at the nodes and pipe diameters are obtained by a slight increase in pump speed (by 3%).

### 5.4. Optimal Pump Speed and Pumping Station Costs for Fixed Water Consumption

The last task of water supply network optimisation shown here is the determination of the speed of pump 1 (again, on the example of the Bibiela pumping station) minimising the pumping station operation costs and providing postulated pressure values in selected network nodes n2 and n29 for a given water consumption. The operating cost of the pumping station is calculated on the basis of the cost of energy consumed by the pump and the cost of water treatment in the Bibiela water intake station.

**Figure 17:**
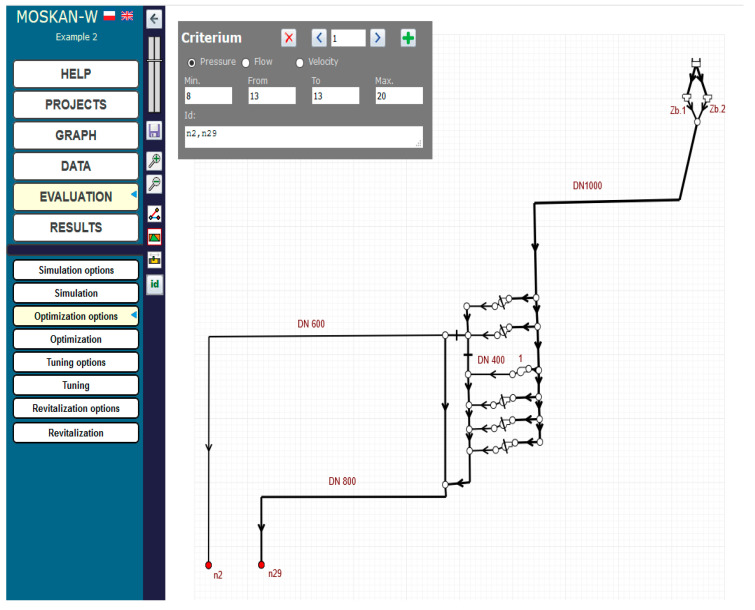
Definition of pressure head criterion for nodes n2 and n29 (Task 5.4).

**Figure 18:**
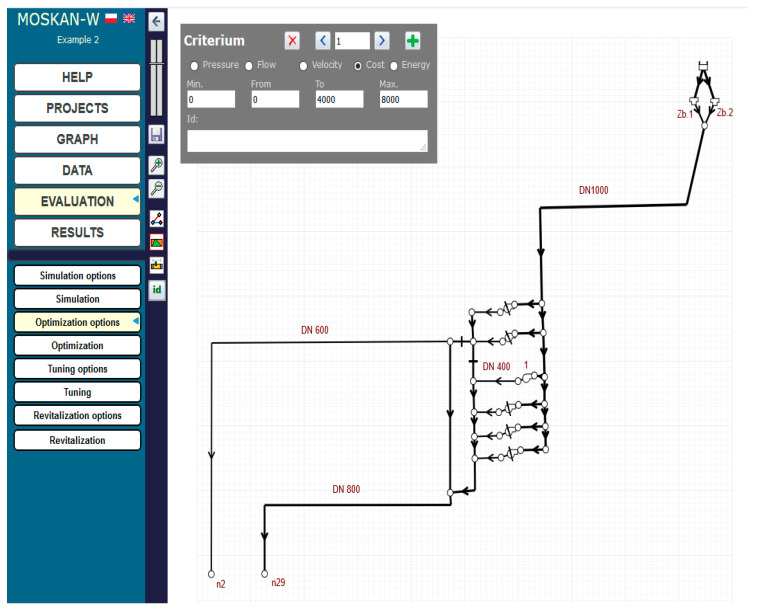
Definition of cost criterion for the pumping station (Task 5.4).

**Figure 19:**
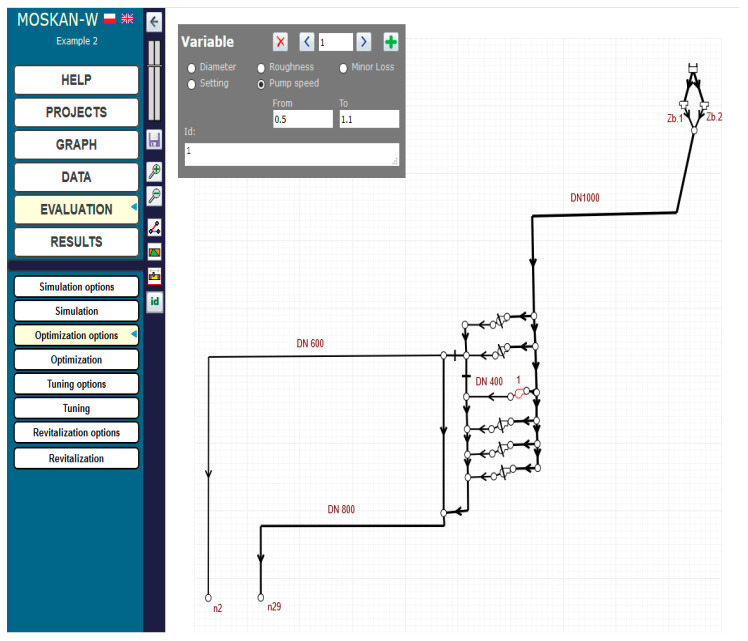
Definition of the control variable (pump l speed) variation range (Task 5.4).

Figure 17 and Figure 18 show the definition of two quality criteria: a single pressure criterion for two nodes n2 and n29 and a cost criterion for the whole pumping station. For the pressure criterion, the range of preferred values is not taken into account, but only a single preferred pressure head value of 13 m, and a range of permissible pressure head values (8–20 m) are defined, whereas for the cost criterion, both the range of preferred values (0–4000) and the range of permissible values (0–8000) are taken into account, while the reported cost values mean hypothetical monetary units. Figure 19 shows the definition of the parameters of the control variable, the pump speed, allowing for its change in the range (0.5–1.1) of the nominal speed.

**Figure 20:**
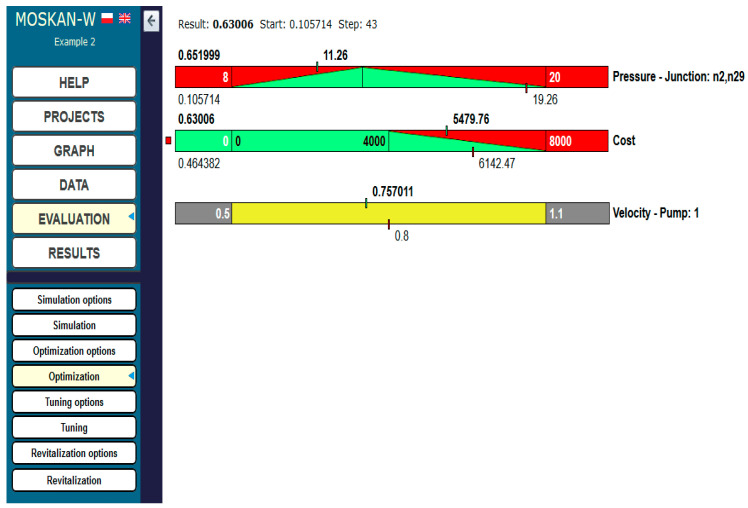
Optimisation results (Task 5.4).

The optimisation results are also shown in Figure 20. An improvement in pressure head was obtained, which significantly approached the preferred value (at the starting point of the calculations, the difference between the current pressure head of 19.26 m and the preferred pressure head of 13 m was about 50%, while after completing the calculations, the difference was only about 8%). At the same time, the operating costs of the pumping station were reduced by approx. 10% with the reduction in pump speed by approx. 6%.

In the examples shown here, the application of optimisation enables us to identify recommendations and suggestions for changes that can be made in various ways, e.g., by changing pump or valve settings, replacing pumps or network elements. When making decisions, it is necessary to take into account various types of restrictions, which are not taken into account when formulating the optimisation task, e.g., availability of facilities with the required technical parameters.

## 6. Conclusions

The presented algorithms have been implemented in an ICT system developed to design, model, and optimise water supply networks. This system supports the comprehensive management of communal water supply networks, and the paper presents solving only tasks related to hydraulic optimisation of the networks. The listed algorithms solve the issues which were formulated as a result of consultations with the waterworks GPW S.A. and seem to be the most pressing problems in the company at present. The developed system is an open system and can be freely extended with other algorithms to solve additional problems related to the management of the waterworks, if they are properly formulated and reported by the water supply company.

In the tasks currently solved by the system, the optimisation calculations use the genetic algorithm and fuzzy set logic to define ranges of variable values in quality criteria. At the same time, algorithms based on neural networks are also being developed, including the use of deep learning methods [36], in particular for the detection and location of hidden leaks on water supply networks, which aims to reduce water losses and, consequently, reduce network operating costs [37,38,39,40,41].

The energy-efficient network operation, reduction in water losses, and improvement of water quality are the three key problems encountered in the operation of urban water supply networks. The developed ICT system generates optimisation scenarios, which may or may not be implemented in practice by network operators. The further development of this system will be aimed at automating the process of exploitation of the water supply network, i.e., on-line control of the operation of executive devices installed on the network [34]. In particular, this concerns the task of controlling the closing of the sliders to increase the flow velocity of water in the network pipes in order to prevent it from becoming corrugated and improve its quality.

It can be noted that work on the results of the implementation of the developed calculation algorithms into the operating practice of real water supply companies is quite rarely referred to. Such few cases are, for example, the works [18,42], which discuss the results of control of water supply networks in Barcelona and Toronto, respectively. In general, algorithms are developed and the results of calculations made on the basis of synthetic data and hypothetical object models are referred to, which, unfortunately, are not transferable to real conditions and usually require large and time-consuming adaptation changes when performing such tests.

From the examples presented in this article concerning the application of optimisation algorithms to control pumps on the water supply network, it can be seen that the possible reduction in energy costs of the pumps are at the level of a few or maximal dozen percent, and also at this level the energy costs increase, if the algorithm shows the advisability of increasing the pumps speed. These observations are important because they are the result of using the developed algorithms in the operation practice of a real water supply company, although similar results were also obtained on the basis of simulation calculations with the use of synthetic data [24].
